# The self-regulation for learning online (SRL-O) questionnaire

**DOI:** 10.1007/s11409-022-09319-6

**Published:** 2022-09-01

**Authors:** Jaclyn Broadbent, E. Panadero, J. M. Lodge, M. Fuller-Tyszkiewicz

**Affiliations:** 1 School of Psychology, Deakin University, Geelong, Australia; 2 Centre for Research in Assessment and Digital Learning, Deakin University, Geelong, Australia; 3 Centre for Research for Educational Impact (REDI), Deakin University, Geelong, Australia; 4 Facultad de Educación Y Deportes, Universidad de Deusto, Bilbao, Spain; 5 IKERBASQUE, Basque Foundation for Science, Bilbao, Spain; 6 School of Education, The University of Queensland, Brisbane, Qld Australia; 7 Center for Social and Early Emotional Development, Deakin University, Burwood, Australia

**Keywords:** Self-regulated learning, Questionnaire, Online learning, Blended learning, Motivation, Learning strategies

## Abstract

The Self-Regulation for Learning Online (SRL-O) questionnaire was developed to encompass the breadth of motivational beliefs and learning strategies that are often used in online and/or blended learning contexts. No current measure meets all these needs. This study used two non-duplicate samples to provide evidence of the psychometric properties of SRL-O using exploratory factor analyses (sample 1, *n* = 313), and confirmatory factor analyses, convergent and content validity and reliability (sample 2, *n* = 321). The SRL-O has a 10-factor structure, made up of (1) online self-efficacy, (2) online intrinsic motivation, (3) online extrinsic motivation, (4) online negative achievement emotion, (5) planning and time management, (6) metacognition, (7) study environment, (8) online effort regulation, (9) online social support, and (10) online task strategies. The SRL-O was also found to have two superordinate factors (motivational beliefs and learning strategies). The SRL-O was demonstrated to be a psychometrically sound measure of online SRL for learners studying in online and blended learning contexts. There is no other online self-regulated learning questionnaire that currently covers such a wide range of motivational beliefs and learning strategies.

## Introduction

The proportion of students undertaking online forms of study has been increasing year on year (National Centre for Education Statistics, 2017, 2018, 2019), with one in three students taking at least one online subject within their degree in the USA (National Centre for Education Statistics, 2019). Online technology is so widely used in higher education in recent times that most classroom instruction would be considered a blended mix of face-to-face instruction with mediating technologies (such as a Learning Management System; Rasheed et al., 2020). Furthermore, with the 2020 pandemic resulting in stay-at-home orders worldwide, most, if not all, current higher education students will have experienced online learning to some degree in what has been called ‘emergency remote teaching’ (Hodges et al., 2020).

A core promise of online learning is the flexibility to study anywhere, anytime. However, this delivery mode may also result in reduced opportunities for interactions with teachers and peers, consequently increasing the need for learners to determine for themselves when and how to engage with learning activities (Broadbent & Lodge, 2020; Kizilcec et al., 2017). Given the high level of autonomy and self-direction that is required with online learning, it is not surprising that self-regulated learning (SRL) plays an essential role in academic success when studying online (Broadbent & Poon, 2015). Importantly, the field is missing a validated instrument to measure students' motivated SRL in an online/blended learning context. A measure of motivated SRL would include both motivational beliefs (such as self-efficacy) and learning strategies (such as metacognition). While self-report measures do have their limitations, self-report has the advantage of being able to be administered to large groups in a cost- and time-effective manner (Jansen et al., 2020; Schellings & Hout-Wolters, 2011), and hence can provide a convenient and potentially useful source of data for understanding student SRL. We thus aim to develop and test the psychometric properties of a newly designed instrument that incorporates both SRL motivations and learning strategies based on students’ self-report.

### Self-regulated learning

Learners differ in the extent to which they use self-regulation by setting goals, planning, and engaging in strategies to achieve their learning objectives. Through evaluation and reflection, learners monitor and modify these strategies to enhance their progress toward goal achievement (Zimmerman, 1986). A successful self-regulated learner is usually oriented towards learning goals, persists when facing challenges, manages their time effectively, and seeks assistance when necessary (Pintrich et al., 1993). Meta-analytic research has shown that SRL strategies are positively related to academic outcomes in primary, secondary, and higher education settings (e.g., Dignath & Büttner, 2008; Richardson et al., 2012; Schneider & Preckel, 2017) as well as in online settings (Broadbent & Poon, 2015). Further, a meta-analytic study on the effects of SRL training demonstrated it could improve academic achievement, motivation and learning strategy use, such as metacognitive and resources management strategies (Theobald, 2021). As students increasingly engage in online or blended learning, either through necessity or choice, we need to continue to work on understanding which SRL strategies are most important and how learners can best apply SRL strategies to achieve academic success within the online environment. Answers to these research questions are contingent upon continuing to refine our measurement tools within this online context.

### Developing a new measure of online SRL

We wanted to develop and validate a comprehensive measure of SRL that included motivational beliefs (such as self-efficacy) and SRL learning strategies (such as metacognition) suitable for online and blended learning contexts. We took a social cognitive perspective, and we drew on essential components of SRL theory that have previously been shown to be important in works by Zimmerman and Moylan (2009) and Pintrich et al. (1993). We believe that any comprehensive SRL questionnaire should include both motivational beliefs and learning strategies (herein called self-regulated motivational and learning strategies). Motivational beliefs are important in the forethought phase of learning and throughout the learning experience, and learning strategies are crucial during the performance phase (Zimmerman & Moylan, 2009). We leveraged expectancy-value theory for the motivational scales, which includes (a) expectancy (beliefs about ability), (b) values (reasons why you want to do the task) and (c) affect (emotional reactions; Pintrich et al., 1991, 1993; Pintrich, 1988, 1989). In deciding, which scales to include for the motivational and learning strategies, we reviewed the current online SRL measures available (see Table 1). We also reviewed the Motivated Strategies for Learning Questionnaire (MSLQ; Pintrich et al., 1991, 1993) due to its popularity and because it is often modified for online and blended learning contexts.

**Table 1:** Comparison of SRL questionnaires

	Self-regulation for learning online (SRL-O; current study)	A measure of SRL used in MOOCs (Kizilcec et al., 2017)	Motivated strategies for learning questionnaire (MSLQ; Pintrich et al., 1991)*	Online academic help-seeking (OAHS; Cheng & Tsai, 2011)	Online learning value and SE scale (OLVSES; Artino & McCoach, 2008)	Online self-regulated learning questionnaire (OSLQ; Barnard et al., 2009)	Online Self-regulation questionnaire (OSRQ; Cho & Cho, 2017)	Online technologies self-Efficacy scale (OTSES; Wang et al., 2013)	Online test anxiety inventory (OTAI; Alibak et al., 2019)	Self-regulated learning efficacy (SrLe) scale (Tladi, 2017)	Self-regulated online learning questionnaire (SOL-Q; Jansen et al., 2017)	Test emotions questionnaire (TEQ; Pekrun et al., 2004)**
Self-efficacy	X		X	X	X			X				
Intrinsic motivation	X		X									
Extrinsic motivation	X		X									
Negative achievement emotion	X		Anxiety						Anxiety			X
Control			X									
Task Value			X		X							
Metacognition	X	Two scales Planning & evaluating	X			Self-evaluation scale	Within student and content scale				X	
Goal setting / planning	X	X				X						
Time management			X			X	X			X	X	
Study environment	X					X					X	
Effort regulation	X		X							X	X	
Social support	X	X	X	X		X	X			X	X	
Task strategies	X	X	X			X						
Cronbach's α***	0.74–0.92	0.75–0.86	0.52–93; 0.67–0.94 (current study)	0.65–0.84	0.88–0.95	0.67–0.90	0.90–0.94	0.95	0.84–0.90	0.83–0.94	0.67–0.90	0.75–0.93

* Not specifically for online and included because of its popularity;** Not specified for online and included because most online SRL measures do not address negative emotions; ***range of Cronbach's α for relevant scales retrieved from original articles; X = included

From our review, the identified online measures included some but not all aspects of SRL. As expected, measures that concentrated on online learning focused on features of the context. For example, the Online Academic Help-Seeking Questionnaire (OAHS; Cheng & Tsai, 2011) incorporates web-based communication tools (such as discussion boards), social media (such as Twitter) and search engines (such as Google) when measuring help-seeking behaviour. The most commonly included subscales for online SRL questionnaires were social support, such as peer learning and help-seeking, time management, environmental structuring (which was sometimes combined with time management), metacognition, and self-efficacy. Metacognition was presented as one scale or as separate subscales of planning, monitoring and/or evaluating. Most of the questionnaires focused on SRL motivational beliefs or strategies, but usually not both (e.g., Barnard et al., 2009; Cho & Cho, 2017; Jansen et al., 2017, 2018; Kizilcec et al., 2017; Tladi, 2017) with the exception of the MSLQ which spanned both strategies and motivations, but it was not designed with the online context in mind.

The Cronbach's α of the relevant scales from each questionnaire ranged from 0.52 to 0.95. The acceptable range for Cronbach's α is between 0.70-0.95, but ideally it should be between 0.70-0.90 (Tavakol & Dennick, 2011). Every questionnaire below has at least one scale that falls outside the 0.70-0.90 range, with the exception of the Online Test Anxiety Inventory (OTAI; Alibak et al., 2019) and a measure of SRL used in MOOCs (Kizilcec et al., 2017). For example, the MSLQ had one scale above 0.90 (self-efficacy) and six subscales that fell below 0.70 (Extrinsic Motivation, Control, rehearsal, organisation, effort regulation and help-seeking), with help-seeking as low as 0.52. The OSLQ (Barnard et al., 2009) had two of its six subscales fall below 0.70 (help-seeking and task strategies). This suggests that there could be issues with the inter-relatedness of items or heterogeneous constructs within some of these scales. Although, it should be noted that scales between 0.90 and 0.95 are still deemed acceptable.

The most commonly used online SRL measure is the Online Self-regulated Learning Questionnaire (OSLQ; Barnard et al., 2009; Roth et al., 2016). This measure contains six strategies (more than most) but no motivational beliefs. Other promising scales have been designed for particular learning environments, such as MOOCs (Jansen et al., 2017, 2018; Kizilcec et al., 2017), which often include learners who have previously completed a post-tertiary degree (DeBoer et al., 2013; Li, 2019), and perhaps more advanced in their learning strategies compared to first-time tertiary learners. While these new measures look promising, to date, no one measure has captured a wide range of learning strategies and motivational beliefs specific to online and blended learning contexts. So, while they are designed with the online student in mind, multiple different measures are needed to cover a wide breadth of strategies and motivations. This is potentially problematic if factors from different questionnaires overlap or lack distinctiveness. The most comprehensive questionnaire is the MSLQ; however, the age of this measure, nearly 30 years, means that changes in how contemporary students study may not be represented satisfactorily (Broadbent, 2017; Broadbent & Poon, 2015; Cho & Summers, 2012). Prior studies (see Artino & Jones, 2012; Broadbent, 2017; Cho & Cho, 2017; Sun et al., 2018) have modified wording to fit the online context, but this hasn't been done in a comprehensive and systematic validation process. These modifications may be sufficient to render a valid measure for an online or blended learning context, but it is not guaranteed. It also assumes that adding a focus to online learning contexts by including phrasing such as "in this online class" is sufficient. While this rewording reorientates the learner to the online context, it is unlikely to capture the breadth of modern activities that learners engage in via online settings. While many of the MSQL questions are relevant today, scales such as test anxiety focus solely on the stress related to tests and exams and thus do not capture the emotional regulation needed for other activities. The peer-learning and help-seeking scales, on the other hand, are limited in scope to the learner's closely located instructor and classmates. Even the addition of "in this online class" to a question misses how students seek help online through a range of tools such as discussion boards, social media, email, and instant messaging. Further, the vast reach of the internet also allows for help from any knowledgeable other, not just limited to peers and teachers in the immediate (physical) vicinity. Hence it is plausible that the meaning of support has shifted in recent times with greater availability of online resources and support structures.

Despite the availability of a range of SRL self-report measures for online/blended contexts, a key gap remains: none incorporate a comprehensive range of motivational, emotional, and learning strategies specifically designed for online and blended learning contexts. The current study aims to develop and validate a measure of online SRL for this purpose. In creating the Self-Regulation for Learning Online (SRL-O) questionnaire, we wanted to develop a psychometrically sound online SRL questionnaire that had a wide breadth of subscales that related to both motivational beliefs and learning strategies; was available in the public domain, was economically feasible to deliver, and could be easily scored; and was designed specifically for undergraduate students. We note that prior attempts to implement the SRL for online purposes has often involved adapting existing items or supplementing existing subscales with dimensions from other measures to better reflect the online context. Thus, to achieve our goal of providing a new and comprehensive measure of online SRL, (a) we consulted several other questionnaires that measured online SRL motivations and/or strategies to ensure that the resulting questionnaire adapted the best attributes from a wide range of measures and did not have a narrow focus on only one (such as the MSLQ); (b) we strategised about what scales should be included, how items should be worded, and length of response scale as a team; (c) we did a content validity check with experts and students, (d) we surveyed students and (e) then we tested the factorial structure through EFA and then CFA on separate samples. We also tested convergent/discriminant validity by exploring the relationship between the MSLQ and the SRL-O.

## Method

### Participants

Participants included 634 students who were randomly split at approximately 50:50 to create two separate samples. Participants came from a university founded in the early 1970s as both a distance and on-campus higher-education provider. The [Anonymous] University is split into four Faculties, which are broad groupings of related discipline areas that we recognise as schools (what others may classify as departments). The four Faculties are: (1) Education and Arts (with schools for Arts; Education; Humanities and Social Sciences); (2) Health (Exercise and Nutrition; Health and Social Development; Medicine; Nursing; and Psychology); (3) Science, Engineering, and Built environment (Architecture, Engineering, Information Technology and Life and Environmental Sciences); and Business and Law (Business and Law). Participants could come from any university course and were not limited to any one course or Faculty. However, based on the authors' advertising reach, it is assumed that a larger proportion came from the Faculty of Health, as well as courses that allow students to study psychology, of which there are many. We recruited a combination of both blended and online learners for two reasons. First, because the University they were recruited from has a strong history of teaching online regardless of enrolment status, and second, the COVID-19 pandemic resulted in learners, regardless of enrolment status, learning either 100% online (or close to) in 2020 and 2021. It was for this second reason we did not separate the two samples by enrolment status.

#### Exploratory factor analyses (sample 1)

Participants were 313 students enrolled in any programme of study at [Anonymous] University. Participants’ ages ranged from 18 to 59 years of age (*M* = 28 years; *SD* = 9 years). This sample comprised 149 blended learners (48%) and 164 online-only learners (52%). The majority of learners were female (82%) and in their first-year of study (40%; second-year 18%; third-year 13%; fourth-year 14%; fifth-year 12%) with a domestic enrolment (90.6%). The majority resided in a metropolitan area (62%; rural remote or regional 38%), were from a medium socio-economic status (72%) and were not the first in their family to attend University (65%). Participants needed to be 18 years or older and currently studying at [Anonymous University].

#### Confirmatory factor analyses and convergent validity (sample 2)

Participants were an unduplicated 321 students enrolled in any programme of study at [Anonymous] University and aged between 18 and 57 years of age (*M* = 29 years; *SD* = 9 years). There were 164 blended learners (51%) and 157 online-only learners (49%). The majority of learners were female (85%) and in their first-year (34%; second-year 19%; third-year 10%; fourth-year 26%; fifth-year 9%) with a domestic enrolment (90.3%). The majority resided in a metropolitan area (69%; rural remote or regional 32%), were from a medium socio-economic status (76%) and were not the first in their family to attend University (61%). There was no significant difference in gender distribution by study mode. Participants needed to be 18 years or older and currently be studying at [Anonymous University].

The two participant groups were found not to differ significantly on age (*t*_(632)_ = 0.41, *p* > 0.05), year level (*t*_(632)_ = 1.64, *p* > 0.05), gender χ^2^ = 2.33, *p* = 0.51), enrolment mode (χ^2^ = 0.77, *p* = 0.38).

### Materials

#### Demographics

Participants reported their (1) age, (2) gender, (3) year level (e.g., year of study in a three or four-year undergraduate bachelor degree or equivalent), and (4) enrolment mode (blended or online).

#### 2.2.2. Motivated strategies for learning questionnaire (MSLQ; Pintrich et al. 1991, 1993)

The MSLQ was used as a measure of convergent validity. The MSLQ consists of 81 items scored on a seven-point rating scale, with defined endpoints of "not at all true of me" and "very true of me". Subscales include motivation components such as intrinsic motivation (α = 0.73) and extrinsic motivation (α = 0.76), control beliefs (α = 0.77), test anxiety (α = 0.85), task value (α = 0.94), and self-efficacy (α = 0.94); metacognitive strategies (α = 0.80) which is a single subscale covering planning, goal setting, task analysis, and self-monitoring; cognitive learning strategies such as rehearsal (α = 0.78), elaboration (α = 0.85), organisation (α = 0.77), and critical thinking (α = 0.88); and resource management strategies such as effort regulation (α = 0.74), time and environment management (α = 0.77), peer-learning (α = 0.73), and help-seeking (α = 0.67).

### Psychometric scale development

Construction of the SRL-O questionnaire proceeded through several key steps as recommended by Devellis and Thorpe (2021). First, several other questionnaires that measured online SRL motivations and/or strategies were consulted as an initial attempt to circumscribe the breadth of SRL in an online context. The measures that were consulted are listed in Table 1.

**Fig. 1:**
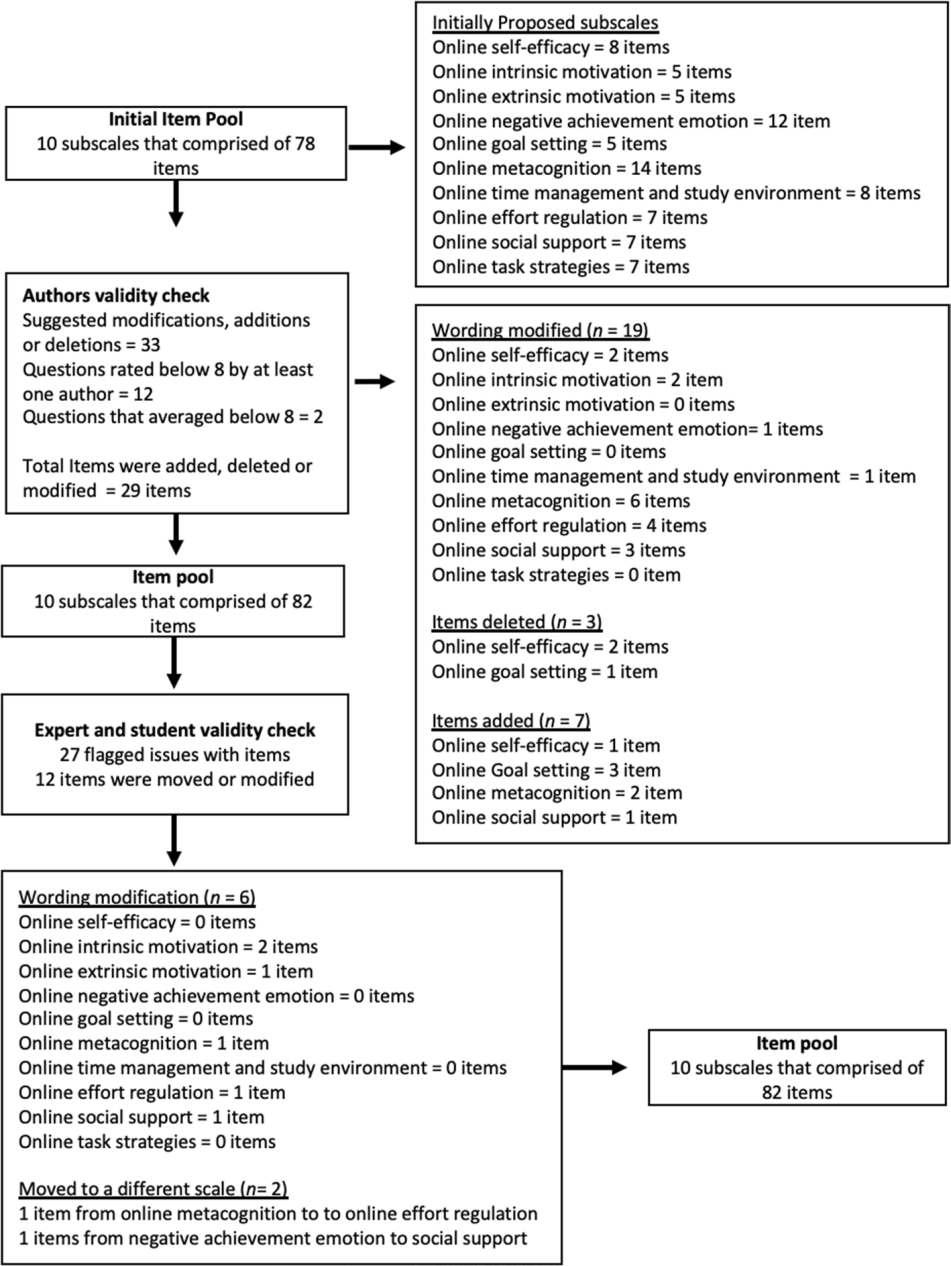
Details of when items were removed, added or modified during scale creation

Second, after a discussion between authors regarding what scales should be included, the lead author designed the questions within each subscale using expert judgement and previous measures as a guide. The initial scale construction consisted of a pool of 78 items made up of ten scales organised into motivational and learning strategies. The initial pool of items was designed to assess SRL comprehensively, so it was expected that there would be some conceptual and statistical redundancy. The lead author also constructed a definition for each scale, as well as a recommendation on how to improve if a learner scored low on the scale. It was decided that all items should be positively worded, as negatively worded items have previously been found to be confusing for participants and create threats to reliability and validity (Chyung et al., 2018; Suárez Álvarez et al., 2018; Van Sonderen et al., 2013). The questionnaire then went to each of the other three authors for review. Authors scored each question out of 10 for representativeness of the scale and made suggestion modifications, additions, and deletions to the items, definitions and recommendations. Final items, definitions and recommendations were then agreed upon by all authors in this step. See Fig. 1 for full details.

Next, the authors decided how many points were to be on the response scale (e.g., 5, 7, 10 or 100 points). The broader scale construction literature was consulted (e.g., Carifio & Perla, 2007, 2008; Jamieson, 2004; Norman, 2010), as well as evaluating what the existing SRL scales had used (see Table 2). Ultimately, it was decided to use a 7-point end-defined response scale with the anchors (1) "not at all very true of me" and (7) "very true of me" at each end and with number labels in the middle (i.e., 2, 3, 4, 5, 6). A 7-point response scale allowed easy comparisons with other popular measures. In designing the online survey, we decided to use radio buttons because visual analogue scales (sliders) have been found to have higher rates of missing data and longer completion times than radio buttons (Couper et al., 2006; Funke, 2016).

**Table 2:** The number of response points and anchors of other online SRL measures

Measure	No. of response points	Negative end	Positive end
OTAI	4	Never	Almost always
OTSES	4	Not Confident	Very confident
TEQ	5	Never	Always
Kizilcec et al.,	5	Not at all true of me	Very true of me
OSLQ	5	Strongly Disagree	Strongly Agree
SRLE	5	Strongly Disagree	Strongly Agree
OLVSES	7	Completely Disagree	Completely Agree
OSRQ	7	Never True	Always True
MSLQ	7	Not at all true of me	Very true of me
SOL-Q	7	Not at all true of me	Very true of me
OAHS	7	Not at all true of me	Very true of me

A content validity check was conducted by sending the questionnaire out to three experts in the field of self-regulation and five students to ask how representative they thought each item was of a particular scale out of 10, with a higher score equally stronger agreement. Each scale provided an opportunity for participants to comment. Any suggested modifications, or questions that scored below 8, were discussed by the authors until consensus was reached (*n* = 27 items). During this process, some items were added, deleted, moved or modified (*n* = 12 items). See Fig. 1. Where items were deemed to overlap, both items were retained so that Exploratory Factor Analyses (EFA) could discern the best item to keep.

Recruitment occurred via online advertisement on course learning management system sites that the authors had access to, student-run University social media groups and by word of mouth. There were no specific follow-up reminders for participating in the study. As the study was advertised broadly across university noticeboards and public forums (e.g., social media groups) that were not controlled by the authors of this study, we were not able to collect or access data on how many students saw the study invitation. Thus, the participant response rate for this study could not be determined. After giving consent, participants completed the demographic questionnaire, the SRL-O, and MSLQ. Participant data were de-identified. The [Anonymous] University ethics board approved the study. Consenting participants were entered into a drawing to win one of thirty $50 gift certificates.

Lastly, the questionnaire is publicly available for download and use at www.srl-o.com. Researchers can also download Qualtrics versions of the questionnaire.

### Data analytic strategy

The overall dataset was split randomly into approximately equal halves to create a subsample of participants (*n* = 313 participants) for testing and refinement of the factorial structure of the measure (often referred to as a training set), and a separate subsample of participants (*n* = 321 participants) to cross-validate the final factor structure obtained from the training subsample (referred to as a test or hold-out set). Given the limited amount of missing data (< 5% across all variables), expectation maximisation was used to impute for missing values (Hair et al., 2010). This evaluation and imputation of missing data, as well as descriptive statistics, correlations, and exploratory factor analysis (EFA), were conducted in SPSS v.26. Confirmatory factor analysis (CFA) was conducted in Mplus v.8.3.

#### Measure testing: Exploratory factor analyses

Several steps were taken to refine the initial pool of 82 items to the finalised item set. First, descriptive statistics were used to identify skew, kurtosis, floor (means < 2, possible score range = 1—7) and ceiling effects (means > 6), and item redundancy (*r*s > 0.8 between items). Second, EFA with maximum likelihood estimation, oblique rotation for potentially correlated factors, and eigenvalues greater than 1 (i.e., Kaiser-Guttman criterion) were used to assess the factor structure of the remaining items. The factor solution was checked for statistically significant cross-loadings (> 0.3 for the present sample size; Hair et al., 2010), items that failed to significantly load onto any factor and item communalities < 0.20 (Hair et al., 2010). We sought factors with three to five items each to balance the brevity, comprehensiveness, and stability of factors. We also evaluated the factor solution to ensure that statistically, defensible factor solutions made sense from a theoretical perspective. This final solution is reported in the Results section.

#### Validation: Confirmatory factor analyses

Our test set was used to validate the factorial solution derived from the steps outlined above. CFA was used for this validation step, and items were set initially to only load onto their primary factor whilst all factors were allowed to covary. Adequacy of model fit was assessed using conventional cut-offs: *p* > 0.05 for chi-square, chi-square/df ≤ 5, CFI ≥ 0.90, and RMSEA ≤ 0.08 (Hair et al., 2010). Modification indices were inspected for sources of model misfit, and theoretically plausible covariances were added to the model as needed to meet acceptable standards of fit. We based this on all fit statistics except for chi-square, which is known to be an overly sensitive measure of fit (DiStefano & Hess, 2005). McDonald’s omega estimates were obtained from this finalised CFA solution to evaluate the internal consistency of subscales. Convergent validity was assessed by correlating these subscales with subscales of an established SRL measure (the MSLQ).

The SRL-O is conceived to have 10 subscales that can be broadly grouped into two superordinate categories (learning and motivation; see Table 3). Hence, researchers may wish to use the scale at the level of the 10 lower order subscales for a detailed profile of student self-regulation or compute the two higher order factors to obtain a smaller number of key factors. Accordingly, we supplement our single-level CFA with a secondary analysis testing the plausibility of a bifactor model in which items load onto the 10 specific factors as well as 2 more global factors of learning and motivation. Fit statistic criteria listed above apply for this secondary analysis.

**Table 3:** Scales from the self-regulation for learning online questionnaire (SRL-O)

	Scale name and definition		Items
Motivational Beliefs	Online self-efficacy	Measures the student’s perceived abilities and belief of academic success in online courses. A high score indicates higher confidence	4
	Online intrinsic motivation	Measures whether the learner perceives themselves to be participating in a task for reasons such as interest, challenge, curiosity, enjoyment, and mastery. A high score indicates higher intrinsic motivation	5
	Online extrinsic motivation	Measures whether the learner perceives themselves to be participating in a task for reasons such as grades, rewards, performance, evaluation by others, and competition. A high score indicates higher extrinsic motivation	3
	Online negative achievement emotion	Includes both negative activating emotions (such as anxiety and shame), as well as negative deactivating emotions (such as hopelessness and boredom). Negative deactivating emotions can have a detrimental impact on motivation, mental processing and increase worry and mental distraction. Negative activating emotions may prompt effort but may also reduce intrinsic motivation and increase ridged strategy use. A high score on this measure indicates a high level of negative achievement emotion	5
Learning strategies	Online planning and time management	Is the structuring of one's efforts toward online study. A high score indicates more planning and time management strategies	5
	Online metacognition	Contains metacognitive planning, monitoring, and evaluating. Online metacognitive planning includes goal setting and task analysis, which makes organising and comprehending material easier. Online metacognitive monitoring includes reflecting, questioning and self-testing as one studies. Online metacognitive evaluating is adjusting and correcting one's cognitive activities and behaviours in response to one's own evaluation of performance during the task. A high score means that one is metacognitively aware while studying	5
	Online study environment	Involves having a study space that is quiet and distraction-free. A high score indicates they can manage the study environment	3
	Online effort regulation	The ability to persist even when the task is uninteresting, there are distractions, or there are more interesting things to do. It requires the learner to be committed to their study goals, control their efforts and implement a range of strategies to do so. A high score means that the learner exerts effort during online studying	4
	Online social support	This scale refers to the learner's willingness to seek help from and collaborate with peers and teachers and through the internet. A high score indicates a greater willingness to seek help and collaborate with others	5
	Online task strategies	Include strategies that help the learner integrate and connect new information with prior knowledge, select appropriate information and also construct connections among the information to be learned, and applying previous knowledge to new situations. A high score on this measure indicates higher task strategy use	5

#### Self-regulation for learning online questionnaire (SRL-O)

The final questionnaire contained 44 items that make up ten subscales measured on a 7-point response scale, with higher scores indicating higher perceived motivation or strategy use. Table 3 shows the name and definition of each scale. The full questionnaire, including scale definitions, recommendations for learners that score under four and scale items, can be found in Appendix Table 8.

## Results

### Measure testing: Exploratory factor analyses

**Fig. 2:**
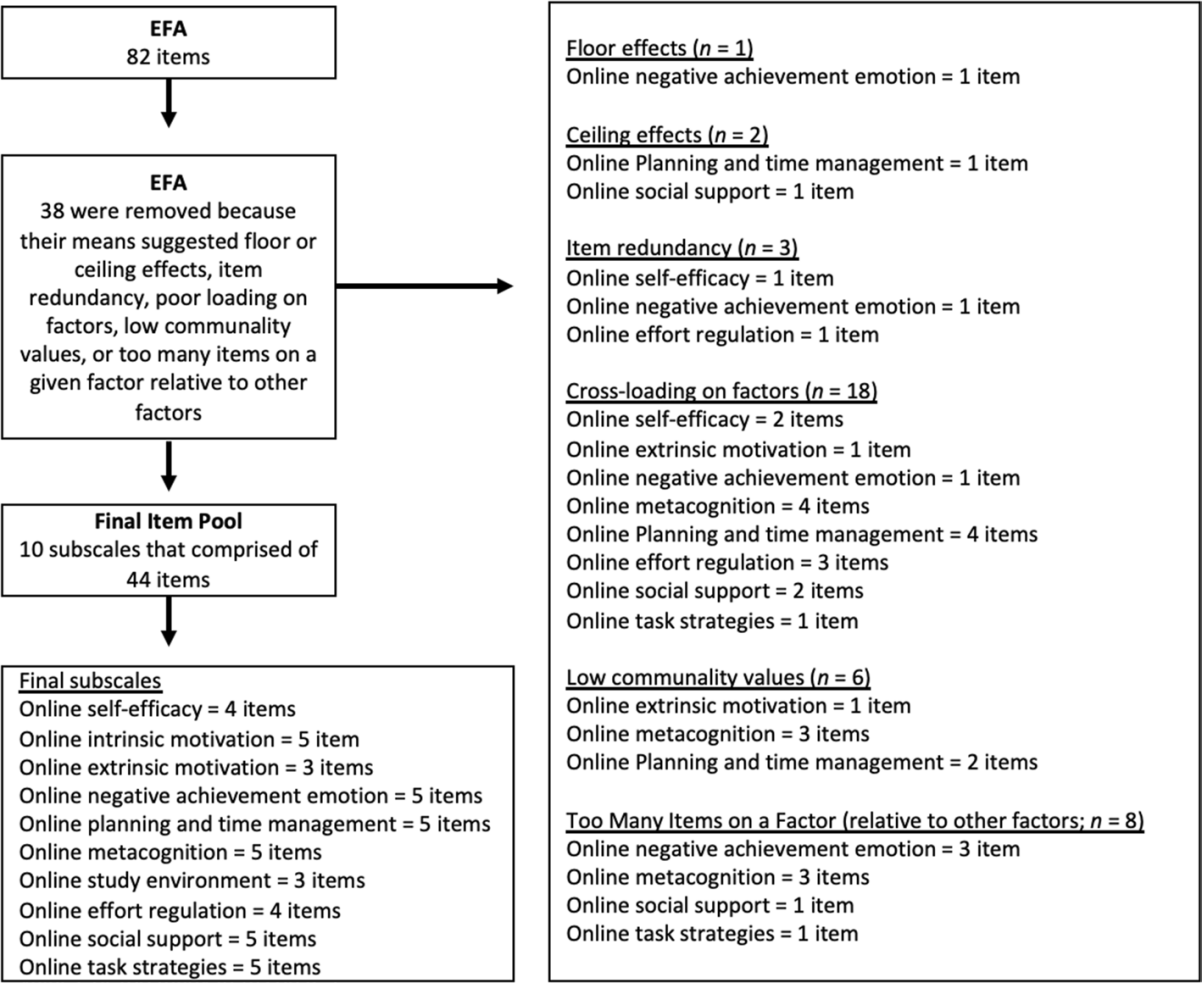
Removed items during EFA

Thirty-eight items (from an initial pool of 82 items) were removed because their means suggested floor or ceiling effects, item redundancy, poor loading on factors, low communality values, or too many items on a given factor relative to other factors (see Fig. 2 for details). Decisions were grounded in an empirical/conceptual basis. The Kaiser–Meyer–Olkin test value of 0.888 and significance of Bartlett’s test (*p* < 0.001) for these remaining 44 items supported the factorability of this item set.

This finalised item set produced the factor structure reported in Table 4 along with factor loadings, mean (SD) and Cronbach's α. In total, these ten distinct factors accounted for 59% of the shared variance among these items. The ten factors represent: (1) online self-efficacy (4 items), (2) online intrinsic motivation (5 items), (3) online extrinsic motivation (3 items), (4) online negative achievement emotion (5 items), (5) planning and time management (5 items), (6) metacognition (5 items), (7) study environment (3 items), (8) online effort regulation (4 items), (9) online social support (5 items), and (10) online task strategies (5 items). The full list of finalised items and their primary factor are listed in Appendix 8.

**Table 4:** Factor loadings from exploratory factor analysis of the SRL-O (*n* = 313)

	Factors										
Item	1	2	3	4	5	6	7	8	9	10	h^2^
SE1	0.740										0.676
SE2	0.670										0.706
SE4	0.649										0.731
SE3	0.557	-0.248									0.708
NE5		0.884									0.855
NE4		0.860									0.815
NE2		0.703									0.633
NE1		0.699									0.670
NE3		0.670									0.631
IM4			-0.802								0.755
IM2			-0.793								0.741
IM3			-0.731								0.774
IM1			-0.612								0.456
IM5			-0.603								0.533
SS5				-0.784							0.662
SS3				-0.777							0.668
SS2				-0.776							0.667
SS4				-0.765							0.624
SS1				-0.576							0.402
EM2					0.774						0.631
EM1					0.709						0.583
EM3					0.627						0.420
SEnvi2						0.827					0.669
SEnvi3						0.780					0.768
SEnvi1						0.407					0.472
P&TM1							0.763				0.690
P&TM4							0.705				0.491
P&TM3							0.648				0.556
P&TM5							0.466				0.500
P&TM2							0.425				0.439
ER1								0.708			0.707
ER2								0.612			0.684
ER3								0.589			0.574
ER4								0.544			0.533
TS1									0.592		0.482
TS5									0.543		0.560
TS6									0.540		0.424
TS2									0.466		0.352
TS3									0.447		0.463
Met5										0.610	0.477
Met4										0.485	0.349
Met2										0.442	0.480
Met3										0.402	0.410
Met1										0.381	0.468
**Mean**	5.19	2.68	5.64	4.44	3.62	4.71	5.20	4.85	4.57	5.48	
**SD**	1.26	1.67	1.15	1.54	1.60	1.48	1.27	1.36	1.18	1.01	
**α**	0.900	0.916	0.877	0.864	0.744	0.774	0.817	0.857	0.774	0.765	

Notes: Factor loadings that are not significant are not shown in the table

*SE/Factor 1* online self-efficacy, *NE/ Factor 2* online negative achievement emotion, *IM/ Factor 3* online intrinsic motivation, *SS/ Factor 4* online social support, *EM/ Factor 5* online extrinsic motivation, *SET/ Factor 6* Study environment, *P&TM/ Factor 7* planning and time management, *ER/ Factor 8* online effort regulation, *TS/ Factor 9* online task strategies, *Met/ Factor 10* metacognition, *h*^*2*^ communality for each item, *α* Cronbach's α

With the exception of one item (Met1on Factor 10), all items loaded > 0.40 on their primary factor. Furthermore, as shown in Table 5, factors had small to moderate inter-relations, suggesting good conceptual separation of these subscales.

**Table 5:** Correlations among factors from EFA (*n* = 313)

Factor	1	2	3	4	5	6	7	8	9
1 (SE)									
2 (NE)	–0.560								
3 (IM)	0.432	–0.424							
4 (SS)	0.243	–0.120	0.245						
5 (EM)	–0.013	0.184	–0.073	–0.020					
6 (SEnvi)	0.421	–0.404	0.287	0.184	–0.031				
7 (P&TM)	0.428	–0.318	0.312	0.217	–0.125	0.396			
8 (ER)	0.582	–0.449	0.512	0.289	–0.140	0.469	0.468		
9 (TS)	0.411	–0.250	0.440	0.315	0.008	0.320	0.417	0.481	
10 (Met)	0.505	–0.252	0.443	0.316	–0.069	0.281	0.481	0.480	0.501

*SE* online self-efficacy, *NE* online negative achievement emotion, *IM* online intrinsic motivation, *SS* online social support, *EM* online extrinsic motivation, *SET* study environment, *P&TM* planning and time management, *ER* online effort regulation, *TS* online task strategies, *Met* metacognition

Correlations >|.120| are significant (*p* < 0.05, two-tailed)

### Validation: Confirmatory factor analysis and reliability estimation

Confirmatory factor analysis for the factor structure derived during measure testing provided inadequate fit initially: χ^2^(857) = 1675.03, *p* < 0.001, χ^2^/df = 1.95, CFI = 0.872, RMSEA = 0.055. Inspection of modification indices identified covariances that could be added to improve fit. In total, seven additional covariances among items were included to achieve acceptable model fit: (1) online intrinsic motivation item 3 (‘*I find studying for this online class enjoyable*’) with online intrinsic motivation item 5 (‘*I get a sense of achievement when I learn new skills or information*’), (2) online negative achievement emotion item 5 (‘*When I have to study online, I start to feel bad*’) with online self-efficacy item 1 (*‘I am confident that I will be able to master the content and assignments in this online class*’), (3) study environment item 2 (*‘I have access to a quiet and distraction-free place to study’*) with study environment item 3 (*‘I know where I can study most efficiently for this online course’*), (4) metacognition item 4 (‘*I look over past feedback I have received and check that I have made improvements in my current learning*’) with metacognition item 5 (‘*I think about how I might improve my work by evaluating it against marking criteria provided by the teacher*’), (5) online task strategies item 3 (*‘When studying online, I try and relate the content to what I already know’*) with online task strategies item 5 (‘I try and improve my understanding by doing additional work beyond the core content (e.g., do extra problem-solving activities or extra readings’), (6) online negative achievement emotion item 3 (‘*While studying, I want to distract myself to lower my anxiety level*’) with online negative achievement emotion item 4 (‘*I get so anxious that I don’t even want to start studying online*’), and (7) online social support item 3 (‘*I ask the teacher and/or my peers to clarify information in my online course*’) with online social support item 4 (‘*When I have difficulties with my online class, I seek assistance from others through online means*’). This revised factor structure had acceptable fit: χ^2^(850) = 1478.31, *p* < 0.001, χ^2^/df = 1.74, CFI = 0.901, RMSEA = 0.048.

Table 6 provides factor loadings, mean (SD), internal consistency estimates and Cronbach's α for these finalised subscales. Nine of the ten factors had internal consistency estimates > 0.70, while consistency was a bit lower for study environment (omega = 0.665), which had three items.

**Table 6:** Factor loadings from confirmatory factor analysis in the SRL-O (*n* = 321)

	Factors										
Item	1	2	3	4	5	6	7	8	9	10	h^2^
SE1	0.756										0.571
SE2	0.814										0.663
SE4	0.820										0.672
SE3	0.739										0.546
NE5		0.811									0.657
NE4		0.779									0.607
NE2		0.809									0.655
NE1		0.830									0.689
NE3		0.835									0.696
IM4			0.661								0.437
IM2			0.809								0.655
IM3			0.875								0.765
IM1			0.821								0.675
IM5			0.751								0.564
SS5				0.614							0.378
SS3				0.797							0.636
SS2				0.775							0.600
SS4				0.781							0.609
SS1				0.834							0.695
EM2					0.810						0.656
EM1					0.807						0.651
EM3					0.508						0.258
SEnvi2						0.722					0.521
SEnvi3						0.470					0.221
SEnvi1						0.708					0.501
P&TM1							0.678				0.459
P&TM4							0.727				0.528
P&TM3							0.769				0.591
P&TM5							0.638				0.408
P&TM2							0.595				0.354
ER1								0.770			0.593
ER2								0.844			0.712
ER3								0.683			0.466
ER4								0.802			0.644
TS1									0.621		0.385
TS5									0.616		0.380
TS6									0.740		0.547
TS2									0.587		0.345
TS3									0.681		0.464
Met5										0.492	0.242
Met4										0.480	0.231
Met2										0.531	0.282
Met3										0.749	0.560
Met1										0.792	0.627
**Mean**	5.208	2.698	5.684	4.444	3.755	4.830	5.160	4.845	4.559	5.472	
**SD**	1.208	1.594	1.120	1.562	1.661	1.423	1.296	1.315	1.179	1.038	
**Omega**	0.864	0.907	0.893	0.873	0.756	0.665	0.810	0.860	0.781	0.729	
**α**	0.883	0.910	0.867	0.865	0.743	0.780	0.811	0.858	0.763	0.758	

Notes: Factor loadings that are not significant are not shown in the table

*SE/Factor 1* online self-efficacy, *NE/ Factor 2* online negative achievement emotion, *IM/ Factor 3* online intrinsic motivation, *SS/ Factor 4* online social support, *EM/ Factor 5* online extrinsic motivation, *SET/ Factor 6* study environment, *P&TM/ Factor 7* planning and time management, *ER/ Factor 8* online effort regulation, *TS/ Factor 9* online task strategies, *Met/ Factor 10* metacognition, *h*^*2*^ communality for each item, *α* Cronbach's α

As a secondary analysis, we evaluated the plausibility of a bifactor structure such that the 44 items of the SRL-O reflect ten lower order factors (as per above) plus two higher order, global factors reflecting that some of these factors tap into a latent variable reflecting learning while the other items reflect a motivation latent variable. The fit of this model was also acceptable; χ^2^(802) = 1435.52, *p* < 0.001, χ^2^/df = 1.79, CFI = 0.901, RMSEA = 0.050. Thus, it seems reasonable for researchers to use either the lower- or higher-level factors for their studies depending on their study aims.

### Validation: Convergent validity

In order to determine the convergent validity of the SRL-O, the MSLQ was used to explore the relationship between the two questionnaires. As expected, Table 7 shows good correspondence between the factors of our new measure and corresponding factors from the MSLQ. For example, the SRL-O self-efficacy scale had a significantly strong positive correlation with the MSLQ self-efficacy scale. Related to both convergent and criterion-related validity, SRL-O negative achievement emotion had a significantly strong positive correlation with MSLQ test anxiety but significant negative correlations with the majority of the other items in the MSLQ as expected.

**Table 7:**
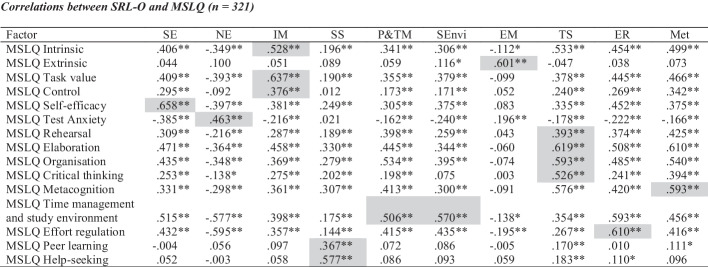
Correlations between SRL-O and MSLQ (*n* = 321)

*Notes*: *SE* online self-efficacy, *NE* online negative achievement emotion, *IM* online intrinsic motivation, *SS* online social support, *EM* online extrinsic motivation, *SET* study environment, *P&TM* planning and time management, *ER* online effort regulation, *TS* online task strategies, *Met* metacognition

*p < 0.05, **p < 0.01 (two-tailed)

## Discussion

This study set out to develop a comprehensive self-report measure of online self-regulated learning (SRL) specifically designed for blended and online learners that incorporated both motivational beliefs and learning strategies. We developed and tested a ten-factor structure of self-regulated learning in online and blended learning environments. The results from the exploratory factor analysis supported our proposed ten-factor solution, and we were able to reduce the size of the measure by nearly half to improve usability. The final factor structure included (1) online self-efficacy, (2) online intrinsic motivation, (3) online extrinsic motivation, (4) online negative achievement emotion, (5) planning and time management, (6) metacognition, (7) study environment, (8) online effort regulation, (9) online social support, and (10) online task strategies. Using confirmatory factor analyses with a non-duplicate sample, we confirmed our ten factors and two superordinate factors (motivational beliefs and learning strategies), and we also provided evidence of convergent validity and internal reliability. Convergent validity analyses showed that scales in the SRL-O correlated with the expected scales in the MSLQ (Pintrich et al., 1991, 1993). For example, SRL-O self-efficacy positively correlated with the MSLQ self-efficacy while negatively correlating with test anxiety, as would be expected. Overall, EFA, CFA, and other validity analyses demonstrate that the SRL-O is a psychometrically sound tool for measuring motivated self-regulated learning strategies for online and blended learners.

The majority of factors that were found in the EFA were reconfirmed in the CFA, with items loading as expected. For example, self-efficacy loaded as a single factor made up of self-efficacy items, intrinsic motivation items loaded with other intrinsic motivation items, etc. Even task strategies, which had previously been found to put the entire factor structure in jeopardy in the development of the SOL-Q (Jansen et al., 2017), were found to coherently cluster together in the current study. The two slight exceptions were for items related to metacognition and time management. While there was an expectation that metacognition might be differentiated on the basis of planning, monitoring and evaluation, metacognition items loaded onto a single factor. This indicates that all metacognitive activities seem to operate together. That is, learners who monitor their progress are also evaluating that progress against a standard or learners who use less monitoring strategies are also engaging in less evaluation. Previous questionnaires have also found metacognition to load as a single factor (e.g., Jansen et al., 2017; Pintrich et al., 1991, 1993).

It was anticipated that items related to time management would form a single factor or combine to form a unique factor with study environment. For example, questionnaires such as the SOL-Q (Jansen et al., 2017) and the OSLQ (Barnard et al., 2009) have found them to be separate factors, while the MSLQ (Pintrich et al., 1991) found these variables associated together. In comparison, the current study found time management combined with planning to make a single factor, while study environment remained a single factor of its own. There is conceptual overlap between planning and time management, so this finding is not surprising, even if it was unexpected. The combined factor makes sense, given that some aspects of planning are used as tools for time management, particularly setting short-and long-term goals, prioritising, making lists, and setting deadlines (Adams & Blair, 2019; Claessens et al., 2007; Macan et al., 1990). Still, it is a scale worth examining again in the future to ensure the combination continues to work together.

### Implications

Our measure of online self-regulated learning adds to the literature in several ways. First, our questionnaire incorporates a range of motivational regulation and learning strategies. Our scale provides four motivational and six learning strategy subscales. Having a variety of subscales may prove useful for different academic outcomes. There is no other online self-regulated learning questionnaire that currently covers such a wide range. Most online SRL questionnaires contain fewer strategies and/or no motivational variables (e.g., Barnard et al., 2009; Cho & Cho, 2017; Jansen et al., 2017, 2018; Kizilcec et al., 2017; Tladi, 2017). The only measure to include such a breadth is the MSLQ (Pintrich et al., 1991), but as argued by Broadbent and Poon (2015) and Broadbent (2017), the MSLQ may not be suitable for online or blended learning.

Second, the SRL-O was shown to have ten subscales that can be broadly grouped into the two superordinate categories of learning and motivation. Confirming subscales and superordinate categories allow researchers to use the scale at the level of the ten lower-order subscales for a detailed profile of student self-regulation or compute the two higher-order factors to obtain a smaller number of key factors. This offers flexibility in how users of SRL-O may choose to report results from the measure. However, we caution that sole use of the superordinate categories may provide an incomplete picture of an individual learner’s SRL profile. We thus encourage researchers to think carefully about the sufficiency of a high-level summary vs a more detailed picture that may be derived from scale totals.

Third, we also decided to include negative achievement emotion. Some measures include test anxiety, but negative achievement emotion is broader as it includes negative activating emotions (such as anxiety and shame), as well as negative deactivating emotions (such as hopelessness and boredom). These items were mostly adapted from Pekrun et al.’s (2011) Achievement Emotions Questionnaire (AEQ), which has been found to negatively predict achievement, in line with previous research that found evidence for the existence of “negative” self-regulation (Alonso-Tapia et al., 2014).

Lastly, we applied a rigorous methodology through our consultation with the literature and internal rating of items by the authors, SRL experts and students. We also consulted the literature around the decision to include a 7-point scale, to only use positively worded items, and to use radio buttons in the online questionnaire. While we tested validity in a variety of ways, future work should also evaluate temporal aspects such as test–retest reliability and sensitivity to change.

### Limitations

This measure is not without its limitations. Learners came from diverse courses across the University. While this helps with the generalisability of our findings, learners from different courses may have different approaches to SRL. In addition, although the measure was only tested on university students, which is a limitation, it would be of interest to explore the use of the measure in different populations such as MOOC or high school students. Further, our study did contain a disproportionately higher number of female participants. While other questionnaires have also had high numbers of female participants (e.g., Cho & Jonassen, 2009; Jansen et al., 2017), a more diverse gender balance should be used in future research. Further, and most importantly, scales in the SRL-O should be analysed in relation to different academic outcomes such as achievement.

Lastly, self-report via questionnaire is one of the most controversial methods to measure SRL (Winne, 2020). Arguably, self-report only measures learners' *perceptions* of the frequency of strategy use, not how successfully the learner implements the strategy – i.e., the "quality" of the implementation (Veenman, 2011; Winne & Jamieson-Noel, 2002). Further, learners may only be able to accurately report strategies they are familiar with or have recently used (Rovers et al., 2019; Winne, 2020). Nonetheless, a number of advantages such as ease of application, interpretation, and ability to reach a large sample size are points often raised in favour of their use (Fryer & Dinsmore, 2020; Pekrun, 2020; Roth et al., 2016). Nonetheless, as argued by Jansen et al. (2020), Jovanović et al. (2017), Zhou and Winne (2012), and Winne (2020), among others, the best approach may be a combined approach that utilises both trace data with survey or interview data, coupled with improving learners’ ability to accurately self-report their learning strategies (Winne, 2020).

### Conclusion

In our study, we aimed to develop a self-report measure of SRL that included both motivations and strategies and could be used in fully online or blended learning contexts. We believe we have successfully achieved this aim and have further contributed to the online SRL literature with a strong instrument that can be used in blended and online learning contexts. This measure has not been tested with students with no online component to their course (e.g. non-blended traditional face-to-face contexts). Nor have single subscales been tested in isolation or in combination with subscales from other measures. Further, some items do not refer to online at all. This opens an interesting empirical question regarding which items do or do not need to mention "online" to capture motivation and learning in online contexts adequately. We would recommend that any adaptation of the questionnaire outside what has been tested here needs to include reliability and validity checks to ensure the questionnaire continues to be psychometrically sound. Importantly, we also want to contribute to the learning community by allowing free access to the questionnaire on www.srl-o.com, which provides automatised scores to the students along with academic recommendations.

## Appendix

Table 8

**Table 8:** Self-regulation for learning online (SRL-O) questionnaire

Name	Online Academic Self-efficacy (Response Scale 1–7)
Definition	Online self-efficacy measures the student’s perceived abilities and belief of academic success in online courses. This scale contains four items. A high score indicates high confidence in mastering class material
Recommendation for those that score under 4	Break tasks into achievable steps so that you can be successful in achieving those steps. Start small, and as you become more successful, make the steps bigger. Look around you at peers and see how they are doing. Can you learn from their approaches? Seek feedback, from yourself and others, as to what you are doing well. Make sure you celebrate your successes
Questions	1. I am confident that I will be able to master the content and assignments in this online class 2. I am confident in my ability to successfully persist in this online class, even if I find the content difficult 3. I am confident I can put in the effort required to get a high grade in this online class 4. I am confident that I can accurately work out what the task is requiring me to do
Name	Online Intrinsic Motivation (Response Scale 1–7)
Definition	Online intrinsic motivation is a measure of the reasons why a learner wants to engage with their learning. In particular, whether the learner perceives themselves to be participating in a task for reasons such as interest, challenge, curiosity, enjoyment and mastery. This scale contains five items. A high score indicates engagement in the task for the sake of learning and not only as a means to an end (such as a grade)
Recommendation for those that score under 4	Online Intrinsic motivation does not come from grades but from your own interest. Reflect on the reasons you originally enrolled in the University. Think about your own personal reasons for learning the material. What do you want to achieve, what do you enjoy learning about, why is it important for you to do well and learn the material? Think about what stimulates your curiosity? Lastly, make sure you celebrate your successes
Questions	1. I always find aspects of the content that arouse my curiosity 2. I love learning new things in this online class 3. I find studying for this online class enjoyable 4. I find it very satisfying when I learn new material in this online course 5. I get a sense of achievement when I learn new skills or information
Name	Online Extrinsic Motivation (Response Scale 1–7)
Definition	Online extrinsic motivation is a measure of the reasons why a learner wants to engage with their learning. In particular, whether the learner perceives themselves to be participating in a task for reasons such as grades, rewards, performance, evaluation by others, and competition. This scale contains three items. A high score indicates engagement in the task as a means to an end (such as a grade)
Recommendation for those that score under 4	Intrinsic motivation is thought to be more helpful than extrinsic motivation. However, you can improve your extrinsic motivation through setting an external goal, such as grade or getting into a postgraduate course
Questions	1. I want to do well in this online course so I can show off to my friends and family 2. I want to do well because of others real or perceived expectations of me 3. I want to get a better grade than others in my online class
Name	Online Negative Achievement Emotion (Response Scale 1–7)
Definition	This measure includes both negative activating emotions (such as anxiety and shame), as well as negative deactivating emotions (such as hopelessness and boredom). Negative deactivating emotions can have a detrimental impact on motivation, mental processing and increase worry and mental distraction. Negative activating emotions may prompt effort but may also reduce intrinsic motivation and increase ridged strategy use. This scale contains five items. A high score on this measure indicates a high level of negative achievement emotion
Recommendation for those that score under 4	If you are feeling anxious or hopeless, take a deep breath and say, 'I can do this', speak to family, friends or a health professional, practice relaxation exercises before studying, and focus on the task, not what others might be thinking, remember times you have performed well in the past. If you are feeling bored, mix up the topics you are studying, reward yourself with regular breaks, or try and make studying fun
Questions	1. I feel so helpless that I cannot dedicate all my effort to my online studies 2. I consider dropping out because I feel overwhelmed by my online studies 3. While studying, I want to distract myself to lower my anxiety level 4. I get so anxious that I don't even want to start studying online 5. When I have to study online, I start to feel bad
Name	Planning and time management (Response Scale 1–7)
Definition	Online planning and time management is about structuring one's efforts and time toward online study. This involves scheduling, planning and setting goals. This scale contains five items. A high score indicates more planning and time management strategies
Recommendation for those that score under 4	Planning and managing time can be long or short term. Think about what you want to achieve from a study session, what you want to achieve from an assignment, and your course. Consider breaking large goals into smaller actionable goals. Consider using a diary with a timetable for weekly planning. Plan out how you meet assignment deadlines across the semester. At the start of each study session, create and prioritise lists of tasks you want to achieve
Questions	1. I set short-term (daily or weekly) goals 2. I set realistic deadlines for learning 3. I break larger goals into smaller actionable goals 4. I make a list of detailed actions that I need to complete 5. I plan out my schedule each week so I have the appropriate amount of time available for online study
Name	Metacognition (Likert scale 1–7)
Definition	Contains metacognitive planning, monitoring, and evaluating. Online metacognitive planning includes goal setting and task analysis, which makes organising and comprehending material easier. Online metacognitive monitoring includes reflecting, questioning and self-testing as one studies. Online metacognitive evaluating is adjusting and correcting one's cognitive activities and behaviours in response to one's own evaluation of performance during the task. This scale contains five items. A high score means that one is metacognitively aware while studying
Recommendation for those that score under 4	Before you start a study session, make a plan of the activities you want to do. Look over the readings/instructions so you get an idea of how it is organised. While looking over the resources, check your understanding of the content or the requirements of the activity. Try to determine which concepts you don't understand well so you can spend more time on them. Ask yourself questions such as, is this task similar to previous tasks? Can I do things differently from last time? Perhaps go back over the old assignment and look at the feedback you have received. How does your performance now compare? Can you adjust your current work based on previous feedback? If available, check your work against the rubric. How does your work compare? Are you meeting the standards you want to achieve?
Questions	1. I think about what learning strategies have worked for me in the past when doing similar assignments/types of study 2. I spend time trying to interpret the task to ensure I understand accurately what I need to do 3. I usually self-assess my performance once I finish 4. I look over past feedback I have received and check that I have made improvements in my current learning 5. I think about how I might improve my work by evaluating it against the marking criteria provided by the teacher
Name	Study Environment (Response Scale 1–7)
Definition	Involves having a study space that is quiet and distraction-free. This scale contains three items. A high score indicates learners can manage their study environment
Recommendation for those that score under 4	Make sure you can find a quiet, distraction-free place to study. You may want to change the place where you study, or the times when you study, or who is around you when you study
Questions	1. I am able to study for my online course without distraction 2. I have access to a quiet and distraction-free place to study 3. I know where I can study most efficiently for this online course
Name	Online Effort Regulation (Response Scale 1–7)
Definition	Online effort regulation is the ability to persist even when the task is uninteresting, there are distractions, or there are more interesting things to do. It requires the learner to be committed to their study goals, control their efforts and implement a range of strategies to do so. This scale contains four items. A high score means that the learner tries hard and exerts effort during online studying
Recommendation for those that score under 4	Keep a list of the topics that you find yourself procrastinating instead of studying. Try to analyse why you postpone studying these topics. Think about the strategies you could use to help you persist. For example, at the start of a study session, make a list of small achievable goals and concentrate on just achieving one at a time. Put distractions such as your phone in the other room. Set yourself a timer to study for a period of time (e.g., 30 min), before stopping for a break. Give yourself a reward if you reach a planned study goal
Questions	1. I work hard in my online study, even when there are more interesting things to do 2. When my online study gets difficult, I remain committed to reaching my study goals 3. When my mind begins to wander during a learning session for this online course, I make a special effort to keep concentrating 4. No matter how I am feeling, I persevere with my online study
Name	Online Social Support (Response Scale 1–7)
Definition	This scale refers to the learner's willingness to seek help from and collaborate with peers and teachers and through the internet. This scale contains five items. A high score indicates greater seek help and collaboration with others
Recommendation for those that score under 4	Consider talking to your teacher, peers in your class, or learning advisors to see how they can help. Connecting with, or learning from, teachers and peers does not have to be synchronous; consider other ways to connect through email, discussion boards and social media. Use online search engines to help you understand the content better
Questions	1. I try to help other students when they ask a question online, I can answer 2. I ask for help from knowledgeable others through online channels when I am not sure what to do in my online class 3. I ask the teacher and/or my peers to clarify information in my online course 4. When I have difficulties with my online class, I seek assistance from others through online means (discussion boards, social media, email, instant messaging etc.) 5. I use email, discussion boards, social media, etc., to connect with the teacher and other students when I need help
Name	Online Task Strategies (Response Scale 1–7)
Definition	Task strategies include strategies that help the learner integrate and connect new information with prior knowledge, select appropriate information and also construct connections among the information to be learned, and apply previous knowledge to new situations. This scale contains five items. A high score on this measure indicates higher task strategy use
Recommendation for those that score under 4	When reading or listing to lecture content, spend time thinking about how the material relates to information you already know. Can you create your own examples that are different from the ones given? Try and make summaries of what you have learnt in your own words. Think critically about what the information means and whether you agree with the author's conclusions
Questions	1. When studying online, I create my own examples of the content to make it more meaningful 2. When studying online, I organise my thoughts by making summaries of what I am learning 3. When studying online, I try and relate the content to what I already know 4. When learning the online content, I try and develop my own ideas about it 5. I try and improve my understanding by doing additional work beyond the core content (e.g., doing extra problem-solving activities or extra readings)
